# Evaluation of drop vertical jump kinematics and kinetics using 3D markerless motion capture in a large cohort

**DOI:** 10.3389/fbioe.2024.1426677

**Published:** 2024-10-24

**Authors:** Tylan Templin, Christopher D. Riehm, Travis Eliason, Tessa C. Hulburt, Samuel T. Kwak, Omar Medjaouri, David Chambers, Manish Anand, Kase Saylor, Gregory D. Myer, Daniel P. Nicolella

**Affiliations:** ^1^ Southwest Research Institute, San Antonio, TX, United States; ^2^ Emory Sports Performance And Research Center (SPARC), Flowery Branch, GA, United States; ^3^ Emory Sports Medicine Center, Atlanta, GA, United States; ^4^ Department of Orthopaedics, Emory University School of Medicine, Atlanta, GA, United States; ^5^ Department of Mechanical Engineering, Indian Institute of Technology Madras, Madras, India; ^6^ Youth Physical Development Centre, Cardiff Metropolitan University, Wales, United Kingdom; ^7^ Wallace H. Coulter Department of Biomedical Engineering. Georgia Institute of Technology & Emory University, Atlanta, GA, United States; ^8^ The Micheli Center for Sports Injury Prevention, Waltham, MA, United States

**Keywords:** markerless motion capture, synthetic data, drop jump, biomechanics, validation

## Abstract

**Introduction:**

3D Markerless motion capture technologies have advanced significantly over the last few decades to overcome limitations of marker-based systems, which require significant cost, time, and specialization. As markerless motion capture technologies develop and mature, there is increasing demand from the biomechanics community to provide kinematic and kinetic data with similar levels of reliability and accuracy as current reference standard marker-based 3D motion capture methods. The purpose of this study was to evaluate how a novel markerless system trained with both hand-labeled and synthetic data compares to lower extremity kinematic and kinetic measurements from a reference marker-based system during the drop vertical jump (DVJ) task.

**Methods:**

Synchronized video data from multiple camera views and marker-based data were simultaneously collected from 127 participants performing three repetitions of the DVJ. Lower limb joint angles and joint moments were calculated and compared between the markerless and marker-based systems. Root mean squared error values and Pearson correlation coefficients were used to quantify agreement between the systems.

**Results:**

Root mean squared error values of lower limb joint angles and joint moments were ≤ 9.61 degrees and ≤ 0.23 N×m/kg, respectively. Pearson correlation values between markered and markerless systems were 0.67-0.98 hip, 0.45-0.99 knee and 0.06-0.99 ankle for joint kinematics. Likewise, Pearson correlation values were 0.73-0.90 hip, 0.61-0.95 knee and 0.74-0.95 ankle for joint kinetics.

**Discussion:**

These results highlight the promising potential of markerless motion capture, particularly for measures of hip, knee and ankle rotations. Further research is needed to evaluate the viability of markerless ankle measures in the frontal plane to determine if differences in joint solvers are inducing unanticipated error.

## 1 Introduction

Analysis of complex human movements provides critical insight across a broad range of health, disease, and performance-related applications. To generate such insights, researchers and clinicians frequently aim to associate both patterns of movement (kinematics), as well as internal and external forces and torques applied to the body (kinetics) with specific health-related conditions (Xu et al., 2023; Xu et al., 2024). Traditionally, accurate and reliable measurement of kinematics through marker-based motion capture requires a dedicated laboratory space with advanced instrumentation operated by highly trained individuals. This process is exceedingly time-consuming and can significantly limit use in routine clinical and functional athletic assessments.

To address these limitations, several markerless motion capture technologies have emerged in recent years. Advancements in computer vision and artificial intelligence, particularly in deep learning-based systems, have shown promise in achieving accuracy levels comparable to marker-based tracking (Kanko et al., 2021; Nakano et al., 2020; Needham et al., 2022). These neural network-based markerless systems rely on large sets of labeled training images, typically obtained from publicly available datasets such as Microsoft Common Objects in Context (COCO) (Lin et al., 2014), Max Planck Institut Informatik (MPII) Human Pose (Andriluka et al., 2014), and Leeds Sports (Johnson and Everingham, 2010) or proprietary datasets (Kanko et al., 2021). While these datasets have facilitated the rapid development of human pose estimation models, the process of manual labeling is labor-intensive and prone to human error. Additionally, many of these datasets offer sparse keypoint labels that limit their utility for comprehensive kinematic and kinetic analyses, particularly for accurately capturing non-sagittal planes of movement (Needham et al., 2022).

One method to overcome the limitations of manual labeling is the use of optical motion capture systems to generate automated 3D labels for training, as demonstrated in HumanEva (Sigal et al., 2010), Human3.6M (Ionescu et al., 2014), and TotalCapture (Gilbert et al., 2019). By projecting 3D labels onto 2D image frames, this technique enables efficient and consistent labeling of occluded points and additional body landmarks. However, motion capture-based datasets often lack the environmental and subject diversity seen in public datasets, which can hinder generalizability. Furthermore, such datasets can suffer from inherent errors associated with marker placement, soft tissue artifacts, and indirect measurement of joint centers, impacting the fidelity of kinematic data.

In response to these challenges, synthetic datasets such as AGORA (Patel et al., 2021), SURREAL (Varol et al., 2017), and Infinite Form (Weitz et al., 2021) have been developed to provide diverse, automatically labeled training images at a large scale. Studies show that incorporating synthetic data into neural network training can reduce error in both 2D and 3D joint position measurements. However, the efficacy of synthetic datasets in reliably producing biomechanics metrics, such as joint angles and joint moments, remains unclear. It is uncertain whether markerless motion capture trained on synthetic data can achieve the necessary accuracy and fidelity across all three planes of motion to evaluate complex dynamic movements.

The drop vertical jump (DVJ) is one example of a complex dynamic movement that is widely used for assessing movement quality, injury risk, and rehabilitation progress through 3D kinematic and kinetic analysis. Numerous studies have characterized the DVJ’s kinematic and kinetic profiles in various populations (Bates et al., 2013; Ford et al., 2003; Ford et al., 2005; Ford et al., 2009; Ford et al., 2010a; Ford et al., 2010B; Hewett et al., 2004; Hewett et al., 2005a; Hewett et al., 2005b; Hewett et al., 2015; Myer et al., 2005; Myer et al., 2013; Myer et al., 2014; Paterno et al., 2010; Pedley et al., 2020). The task has shown high within-session reliability for kinematic and kinetic measures at the hip, knee, and ankle (interclass correlation coefficients (ICC): 0.78–0.99). Although reliability decreases slightly over time in young athletes (ICCs 0.60–0.92 and 0.59–0.87, respectively) (Ford et al., 2007), the DVJ remains a robust tool for comparative analysis of motion capture methods. Its reliability and standardization have led to its inclusion in lab-based motion capture and markerless clinical prediction models aimed at assessing injury risk (Myer et al., 2011). Therefore, the DVJ is an ideal movement task for comparing marker-based and markerless motion capture systems.

Thus, the purpose of this study was to evaluate 3D kinematics and kinetics calculated from a markerless motion capture and compare to kinematics and kinetics obtained from a marker-based system for the DVJ. We hypothesized that the markerless system, trained with synthetic data, would produce 3D kinematic and kinetic measures that closely align with marker-based data, particularly in non-sagittal planes of motion, due to the enhanced representation of these planes of motion provided by synthetic datasets.

## 2 Materials and methods

### 2.1 Marker-based motion capture data collection

This study includes a retrospective analysis of DVJ biomechanics data collected as part of a large prospective study meant to assess ACL injury risk (U01AR067997). Data were collected from 127 adolescent female athletes [age (average ±1 standard deviation) 15.56 ± 1.38 years (range:12–18 years), height: 1.66 ± 0.07 m, weight: 64.40 ± 12.28 kg, race/ethnicity: American Indian/Alaskan Native = 0, Asian = 5, Black/African American = 9, Hispanic = 20, Non-Hispanic White/Caucasian = 78, Native Hawaiian/Other Pacific Islander = 1, More than one race = 11, Declined to answer = 2]. All participants or their legal guardians (if < under 18 years of age) provided informed consent to participate in this study according to an International Review Board protocol approved by Emory University (STUDY00001770). Over the course of 5 weeks, athletes in this sample participated in a large prospective study meant to assess ACL injury risk, featuring the DVJ task. In this protocol, each participant performed three repetitions of the DVJ. The DVJ consisted of the subjects starting on top of a 31 cm box with their feet positioned 35 cm apart and arms held comfortably at their sides. They were instructed to jump slightly from both feet as to drop directly down off the box and immediately perform a maximum vertical jump towards an overhead target, raising both arms as if they were jumping for a basketball rebound (Ford et al., 2007).

Video-based markerless cameras and a marker-based motion capture system (both Qualisys AB, Göteborg, Sweden) were used to capture movement data. The marker-based system used a set of 55 retroreflective full-body markers to capture the DVJ movement (DiCesare et al., 2019). Markers were placed on the thigh, shank, foot, and upper arm forearm, with a focus on achieving high accuracy in lower extremity tracking. Before performing any movements, a static trial was captured with the participant in a T-pose. The markerless setup used 12 Qualisys Miqus cameras recording at a frequency of 120 Hz, while optical marker-based motion capture was collected using 80-Qualisys cameras at 240 Hz. Two force plates (AMTI; Advanced Medical Technology, Inc., Watertown, MA) were located in the center of the capture volume and recorded ground reaction forces (GRF) and center of pressure at 1,200 Hz. Qualisys Track Manager software (Qualisys AB, Göteborg, Sweden) calibrated the global 3D reference frame for the markerless video system and force plates such that they coincided with the marker-based global 3D reference frame and has built-in functionality to synchronize the data streams in time.

### 2.2 ENABLE: training data and functionality

The ENABLE (Engine for Automatic Biomechanical Evaluation) markerless biomechanics system (Southwest Research Institute, San Antonio, TX, United States) was used to process video data. ENABLE uses a convolutional neural network to predict the location of 85 keypoints, each with specific and consistent anatomical locations on the human body within each camera view. The core neural network is trained on ∼200,000 labeled images from three broad training data classes: public datasets (∼50,000 images), auto-labeled images from an optical motion capture marker-based system (∼50,000 images), and a custom synthetic dataset (∼100,000 images). The public images consist of select images from the COCO foot, MPII, and Leeds Sports datasets. The MPII and Leeds Sports training sets are labeled with 15 points including joints centers at the hip, knee, ankle, shoulder, elbow, wrist, and head keypoints; the COCO foot dataset includes these as well as additional foot keypoint labels (Cao et al., 2018).

The optical training dataset obtained during marker-based motion capture data collection consists of images of individuals performing various functional and sports-related movements. Images are labeled using a patented approach that identifies 85 3D anatomical locations based on model pose estimations which are reprojected into a 2D space (Templin et al., 2023). The 85 keypoints consist of the same 25 keypoints used in the COCO dataset plus additional keypoints throughout the body to ensure that each of the following body segments has at least three attached to them: head, torso, upper arms, forearms, hands, thighs, shanks, and feet. This allowed for full characterization of six degrees of freedom for each segment.

**FIGURE 1:**
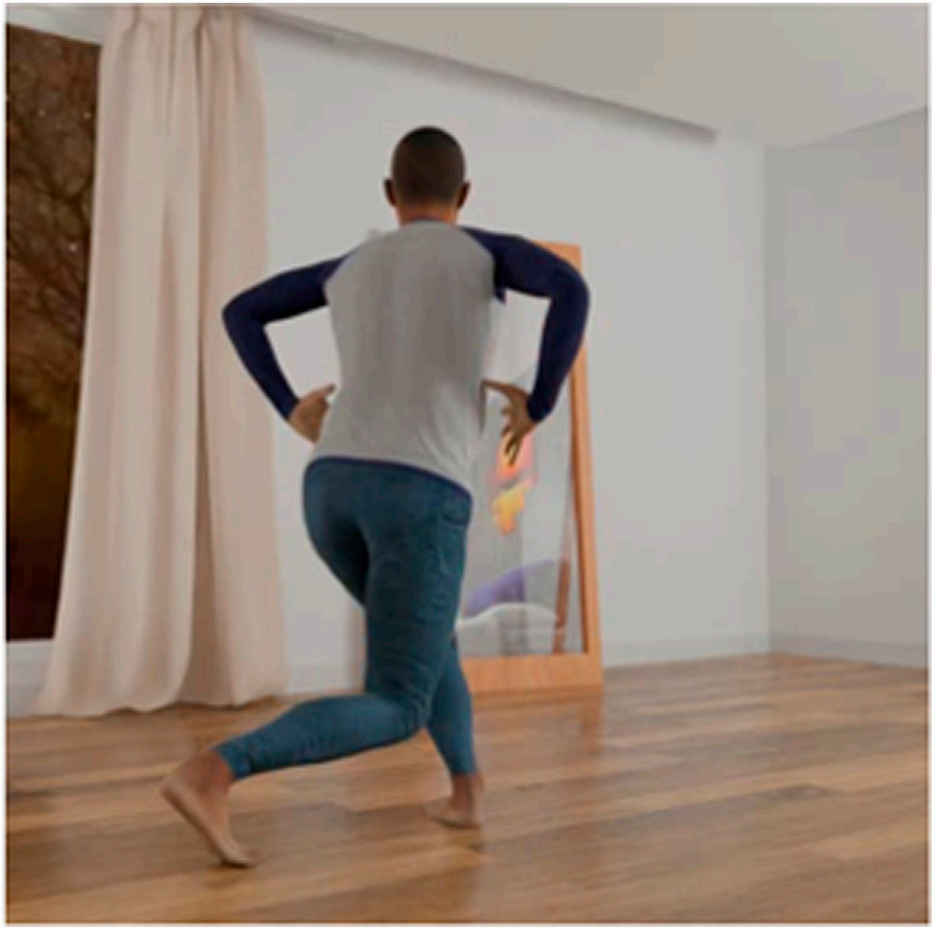
Example image from synthetic dataset.

A custom synthetic dataset with 100,000 images generated by Infinity AI, a synthetic data generation company, is also included in the training dataset, following the approach in the previous work (Weitz et al., 2021). In summary, this dataset consists of synthetic avatars performing various functional movements with varying body size, shape, clothing, skin complexion, camera position, appearance, lighting conditions, and background. Each synthetic image contains an avatar based on the SMPL-X body model (Pavlakos et al., 2019). Panoramas with varied lighting conditions are used as image backgrounds to reflect varying conditions observed in real-world settings. Each of the >10,000 vertices of the SMPL-X model is mapped to a 2D pixel location on the image, and the 85 vertices that most closely correspond to 85-point virtual marker set described above are used as training labels (Figure 1).

During training, the CNN uses each input image to produce a collection of probability maps which correspond to each anatomical landmark and represent the likelihood the landmark is present in any given pixel of the input image. The architecture of the network utilizes multiple concurrent branches at different resolutions that connect at several stages to capture fine-grain and coarse-grain features. This allows the network to correctly locate smaller landmarks such as within the hand while preserving its ability to represent larger relationships such as the ones between limbs. A mean-squared error cost function is used to measure error between the output of the neural network and the ground truth landmark locations to update the network weights. The neural network was trained until the performance on the validation set converged.

Based on the weights established during training, ENABLE identifies the highest probability pixel for each landmark in each frame of the input synchronized videos. Following the 2D detection, a triangulation procedure was used to estimate the 3D position of each anatomical location in each frame. In this process, calibrated camera positions are used to create a set of rays from each camera through each predicted of the 2D keypoint locations. A random sample consensus approach (Fischler and Bolles, 1981) is used to determine a set of inlier and outlier rays. Subsequently, the estimated 3D point is determined to be the point closest to the set of all lines using a least squares approach. The 3D points are then used to scale and generate kinematics for a musculoskeletal model in OpenSim [version 4.4, (Delp et al., 2007),]. The Rajagopal model (Rajagopal et al., 2016) was modified such that it had a total of 14 degrees of freedom in the lower body: 3 hip, 3 knee, and 2 ankles. Ankle subtalar angle includes both transverse and frontal plane motion but for reporting purposes are included in the ab/adduction angles. This model is scaled to match each participant’s anthropometry by using median segment lengths of each segment during each trial as determined by keypoint locations. After the model is scaled, the pose of the model in each frame is globally optimized using inverse kinematics via the Python Application Programing Interface with the OpenSim Inverse Kinematics (IK) tool.

### 2.3 Marker-based and markerless motion capture processing

All marker-based motion capture data were quality controlled, and any gaps in marker tracking were corrected. Next force plate data were filtered with a 4th-order bidirectional Butterworth filter with a 50 Hz low-pass cutoff frequency for further processing (Roewer et al., 2014). Marker and markerless data were filtered with a 4th-order bidirectional Butterworth filter with a 6 Hz low-pass cutoff frequency in Python (version 3.7). Filtered data were used as input for the IK tool in the scaled model as described above. The kinematic output from both markerless and marker-based systems was used as input along with the GRF collected from force plates to calculate joint moments using the OpenSim Inverse Dynamics (ID) Tool. Each trial for both systems was normalized to 101 points during the stance phase of the DVJ. The beginning of the stance phase was determined by the first timepoint when either force plate recorded a measurement of >10 N, and the end of the stance phase was determined by the first timepoint after the starting point when both force plates recorded a measurement of <10 N. The 10 N threshold was used to identify the start and end of the stance phase because it represents a point where the GRF becomes minimal.

### 2.4 Statistical analysis

To evaluate performance of the ENABLE markerless system, IK and ID results for the hip, knee, and ankle were compared to IK and ID results from the marker-based system. Comparisons were made using root mean squared error (RMSE), normalized root mean squared error (NRMSE), and Pearson correlations to quantify the average difference, relative difference and similarity of waveform respectively across the entire time series for each repetition of the DVJ. Pearson correlation strength was defined as the following: 0–0.4 indicates a weak positive correlation, 0.4–0.7 indicates a moderate positive correlation, 0.7 to 0.9 indicates a strong positive correlation, and 0.9–1.0 indicates a very strong correlation (Schober et al., 2018). NRMSE values were calculated for each DOF by normalizing the calculated RMSE by the range of motion of that DOF during the DVJ. For the kinematic RMSE evaluation, a “good” fit was defined as an RMSE value ≤5° (Song et al., 2023), a “moderate” fit was defined as an RMSE value that was ≤10°, and a “poor” fit was defined as an RMSE value that was >10°. For the kinetic comparison, without an established consensus in the scientific community, “good” fit was defined as an RMSE value ≤0.15 N⋅m/kg degrees, a “moderate” fit was defined as an RMSE value that was ≤0.30 N⋅m/kg, and a “poor” fit was defined as an RMSE value that was >0.30 N⋅m/kg degrees.

## 3 Results

### 3.1 Kinematics

**FIGURE 2:**
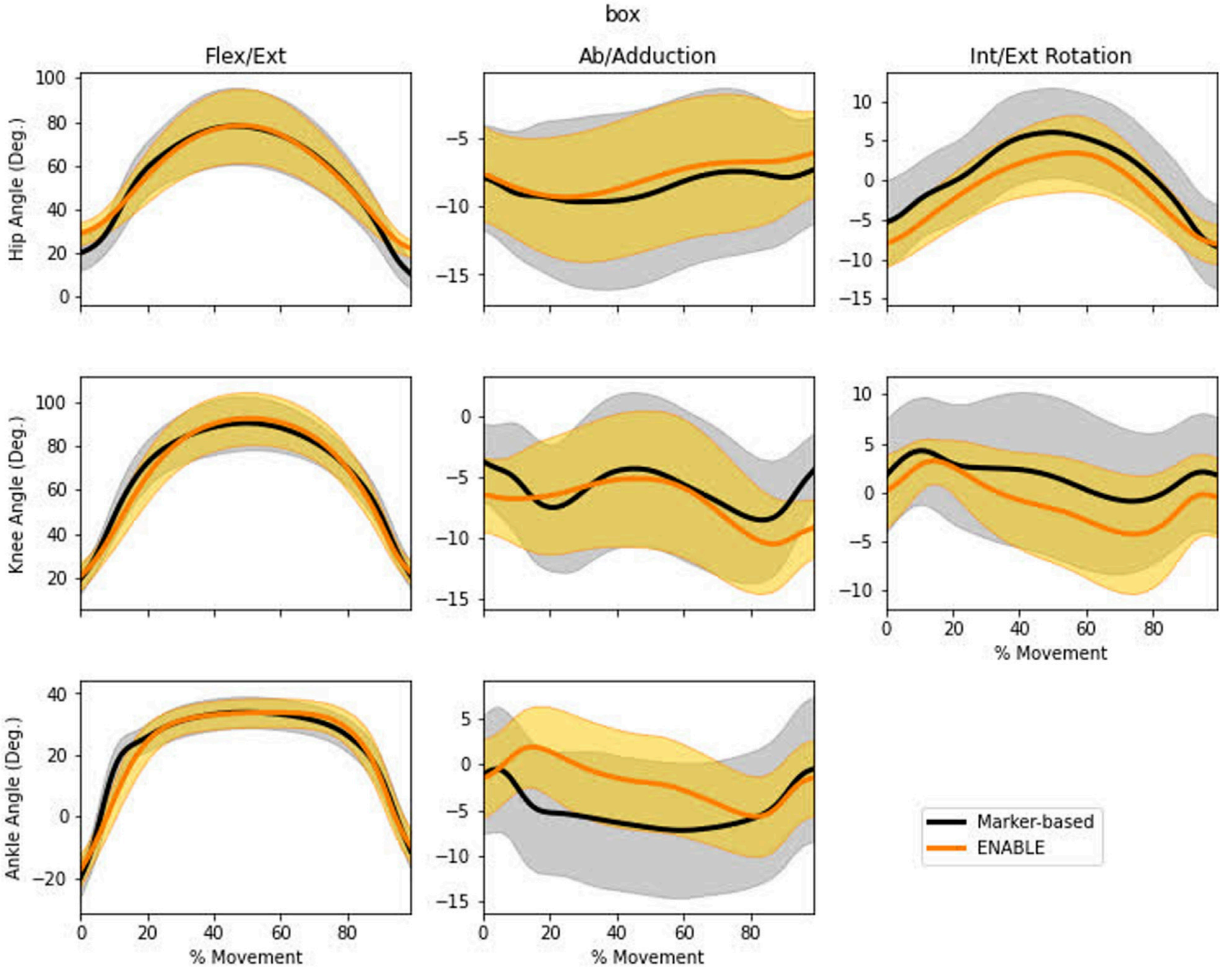
Mean ± 1 standard deviation of the time history trajectories of lower-limb DVJ kinematics measured by the marker-based (black) or markerless (orange) system. Ankle Ab/Adduction is the subtalar angle which includes both transverse and frontal plane motion.

Markerless kinematics demonstrated good to moderate agreement with marker-derived data (Figure 2), with average RMSE values of 2.52–9.21° for hip, knee, and ankle angles (Table 1). Sagittal plane hip, knee, and ankle values had good to moderate fits with RMSE (≤6.93°) and NRMSE (≤0.11) and very strong correlations (≥0.97). Frontal and transverse plane knee and hip kinematics also demonstrated good to moderate fits with RMSE values (≤5.68°), NRMSE values (≤0.66), and moderate correlations (0.45–0.69). The ankle subtalar angle had a moderate RMSE (9.61), the highest NRMSE (0.99), and the lowest correlation value (0.06).

**TABLE 1:** RMSE ±1 standard deviation and Pearson correlation ±1 standard deviation of markerless relative to marker-based system for lower limb DVJ kinematics.

Joint	Degree of freedom	Kinematics		
		RMSE (std) degrees	NRMSE (std)	Pearson (std)
Hip	Flex/Ext	6.93 (0.27)	0.11 (0.05)	0.98 (0.02)
	Ad/Ab	2.52 (1.2)	0.34 (0.22)	0.69 (0.34)
	Int/Ext	5.82 (2.45)	0.40 (0.28)	0.67 (0.29)
Knee	Flex/Ext	5.55 (1.55)	0.08 (0.03)	0.99 (0.01)
	Ad/Ab	4.75 (2.15)	0.54 (0.36)	0.45 (0.43)
	Int/Ext	5.86 (1.90)	0.66 (0.65)	0.59 (0.37)
Ankle	Flex/Ext	5.77 (1.79)	0.11 (0.03)	0.97 (0.01)
	Subtalar	9.61 (4.72)	0.99 (0.66)	0.06 (0.48)

### 3.2 Kinetics

**FIGURE 3:**
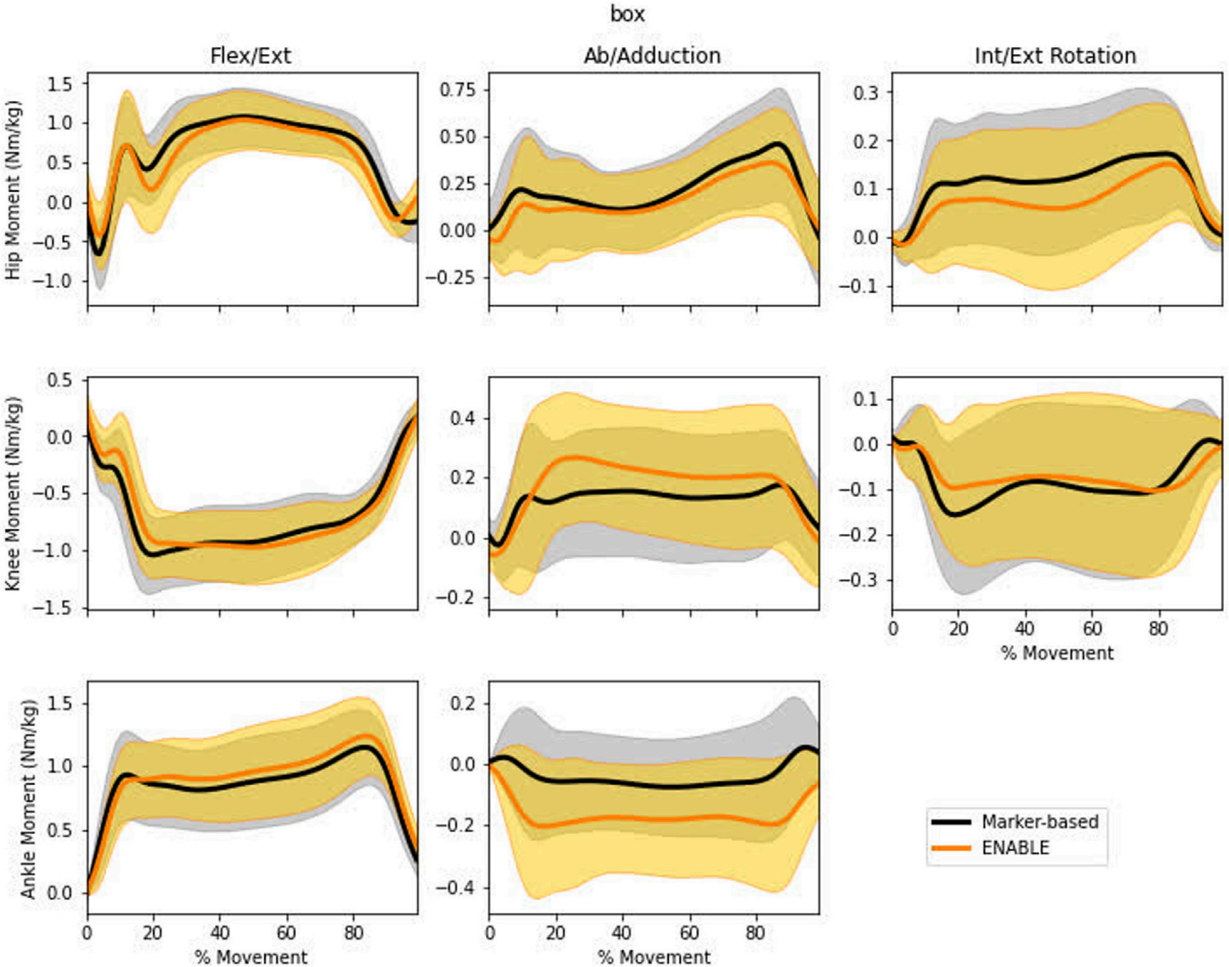
Mean ± 1 standard deviation of the time history trajectories of lower limb DVJ kinetics measured by the marker-based (black) or markerless (orange) system. Ankle Ab/Adduction is the subtalar angle which includes both transverse and frontal plane motion.

Very strong to moderate agreement was observed for hip, knee, and ankle moments (Figure 3). Very strong correlations (≥0.90) were observed for the sagittal plane hip, knee, and ankle moments (Table 2). Strong correlations (0.71–0.78) were observed in the transverse and frontal plane hip, transverse plane knee, and subtalar ankle joint moments. While the frontal plane knee moment exhibited a moderate correlation (0.61). Moderate agreement with respect to RMSE was observed in hip sagittal (0.23 N⋅m/kg), hip frontal (0.17 N⋅m/kg), and knee frontal (0.16 N⋅m/kg) plane moments. All other degrees of freedom showed good agreement (RMSE values ≤0.15 N⋅m/kg).

**TABLE 2:** RMSE ±1 standard deviation and Pearson correlation ±1 standard deviation of markerless relative to marker-based system for lower limb DVJ kinetics.

Joint	Degree of freedom	Kinetics		
		RMSE (std) N⋅m/kg	NRMSE (std)	Pearson (std)
Hip	Flex/Ext	0.23 (0.07)	0.14 (0.04)	0.90 (0.07)
	Ad/Ab	0.17 (0.11)	0.23 (0.17)	0.73 (0.22)
	Int/Ext	0.08 (0.07)	0.30 (0.29)	0.78 (0.21)
Knee	Flex/Ext	0.14 (0.05)	0.09 (0.03)	0.95 (0.02)
	Ad/Ab	0.16 (0.13)	0.42 (0.38)	0.61 (0.32)
	Int/Ext	0.11 (0.09)	0.36 (0.31)	0.71 (0.33)
Ankle	Flex/Ext	0.14 (0.08)	0.11 (0.07)	0.95 (0.04)
	Subtalar	0.15 (0.14)	0.65 (0.6)	0.74 (0.28)

## 4 Discussion

The present study is the first peer-reviewed evaluation of the ENABLE markerless motion capture system compared to a marker-based system. ENABLE has several distinct characteristics relative to other markerless systems. Firstly, the dataset used to train the underlying neural network includes 85 keypoints, with at least three keypoints on each body segment, which is essential for non-sagittal plane tracking. Furthermore, roughly half of the training set consists of synthetic data which be generated quickly and without human intervention, making it highly efficient and scalable. Finally, ENABLE uses musculoskeletal modeling to biomechanically constrain the kinematic output to be physiologically reasonable.

The purpose of the study was to assess the 3D kinematics and derived kinetics from ENABLE relative to a traditional marker-based system. Strong correlations were observed in hip (flexion/extension, ab/adduction, and internal external rotation planes), knee (flexion/extension and internal external rotation), and ankle (plantar/dorsiflexion and subtalar angle) joint kinetics and hip, knee, and ankle sagittal plane kinematics. While moderate associations were observed with knee ab/adduction and internal rotation angles and only subtalar ankle angle had a weak association.

The 127 participants analyzed in this study represent one of the largest cohorts used to estimate performance of a markerless system compared to a marker-based system. Previous studies have focused on cohorts of ≤30 participants (Ito et al., 2022; Kanko et al., 2021; Nakano et al., 2020; Needham et al., 2022; Strutzenberger et al., 2021; Uhlrich et al., 2023) yet a larger sample size considerably improves generalizability of the results. In agreement with previous research (Song et al., 2023), sagittal plane joint angles showed the highest correlation values. However, our hip flexion results were more accurate than results from previous studies reporting mean difference of >22° for squats (Ito et al., 2022) and >16° for countermovement jumps (Strutzenberger et al., 2021). One possible explanation for this finding is that the markerless system identifies points on the pelvis, which many markerless systems are unable to track due to the sparse labels of many commonly used datasets. Consistently, one study (Uhlrich et al., 2023) showed that adding additional anatomical markers (including pelvis points) to a markerless system reduces hip flexion RMSE values by an average of 8.5°. The difference between the present study and Uhlrich et al. is that all our landmarks were inferred directly from the video, while Ulrich et al. created a separate neural network to identify additional landmarks exclusively from sparse keypoint labels.

While RMSE values in the frontal and transverse plane at the knee and ankle were low for the markerless system, these degrees of freedom still exhibited the greatest discrepancy in correlation and NRMSE values with the marker-based data. One potential cause of the decreased accuracy in non-sagittal planes of motion at the knee and ankle is error propagation to distal segments of the kinematic tree (Needham et al., 2022). Since the IK solver used in OpenSim is a global optimization procedure, error observed in the joint may be compounded by error in joints that are more proximal to the root joint (pelvis). This helps explain why NRMSE values increased and Pearson correlations decreased in the non-sagittal plane for joints further down the kinematic chain from the pelvis (i.e., hip correlations > knee correlations > ankle correlations). In addition, including synthetic data into the training pipeline offers many advantages (cost-effective, diverse, scalable, and automatic generation of labeled images), however synthetic data may not capture all the nuances, lighting conditions, textures, and environmental variables present in real-world images. For example, while these data may effectively capture variances in skin tone, differences in lighting can impact how light reflects off each individual’s skin, affecting image segmentation contouring (Sigal et al., 2004). It is possible that this combined variation from lighting and skin tone could alter accuracy of landmark identification and requires future study. Due to the retrospective nature of this study, the lighting conditions, movement type, background, and clothing were not systematically varied. In addition, the racial and ethnicity data collected did not include information about light or darkness of skin tone and, therefore, may not provide enough information to verify that skin tone differences do not impact data accuracy. Our cohort included only adolescent female athletes, so the performance of the current model for other individuals remains unknown. Future studies should investigate the performance of this markerless systems across a wider range of ages, body morphologies, movements, and background environments. A key limitation to the kinetic analysis in this study is the GRF were measured through lab-based force plate equipment. Consequently, despite the portable nature of the kinematic analysis, the kinetic analysis described in this study is tied to the lab. However, novel portable 3D force plates have shown excellent agreement with lab-based equipment (Miller et al., 2023), and alternatively neural network-based GRF predictions from motion capture data (Alcantara et al., 2022; Johnson et al., 2018; Louis et al., 2022) have also shown promising potential to remove the need for lab-based force sensing equipment.

While this study compared the markerless system to the standard of a reference marker-based system, there are well-documented limitations of marker-based systems that can result in erroneous measurements. These include skin motion artifact, marker placement error, and challenges associated with estimating joint centers from markers placed externally to the body (Benoit et al., 2015; Benoit et al., 2006; Cappozzo et al., 1996; Gorton et al., 2009; Kessler et al., 2019; Leardini et al., 2005; Lucchetti et al., 1998; Miranda et al., 2011; Miranda et al., 2013; Peters et al., 2010; Reinschmidt et al., 1997). In addition, marker-based systems also require substantial manual effort to post-process and gap-fill missing marker trajectories. Analogous manual editing of the markerless points was not used to augment the ENABLE data processing, but in the future could be an added feature for users who are experienced with such data post-processing and would like to further improve accuracy. Despite the limitations of marker-based tracking, it is considered the standard to acquire accurate non-invasive kinematic information in the biomechanics community due to the difficultly of collecting data using more accurate methods such as biplane fluoroscopy or the use of bone pins. These methods are often discouraged from use because biplane fluoroscopy uses excessive radiation to capture full-body motion and bone pins are invasive and may impede motion due to discomfort. Our hope is that the results of this study may help to introduce the biomechanics community to markerless tracking methodologies, which have a desirable ease of use at the time of data capture.

In this study we found that the 3D lower limb joint moments derived from the markerless system exhibited similar trends and magnitudes as moments derived from the marker-based system for a DVJ. A few previous studies have estimated joint moments from markerless motion capture data, but 3D analysis of joint kinetics at the hip, knee, and ankle has only been conducted during running (Kanko et al., 2024). Two additional studies investigated joint kinetics with input from markerless motion capture but did not report findings for non-sagittal knee and ankle angles (Song et al., 2023; Tang et al., 2022). Thus, to our knowledge the present study is the first to compare 3D kinetics derived from markerless and marker-based system for a landing and jumping task. In agreement with previous studies, we found that sagittal plane hip, knee, and ankle moments were strongly correlated between markerless and marker-based inputs. In each non-sagittal degree of freedom, the joint moment correlation was greater than the correlation observed for the corresponding joint angle. Despite the trend observed with non-sagittal kinematic data, non-sagittal plane correlations did not show a reduction with increasingly distal joints but instead showed moderate to strong correlations. This finding demonstrates that despite low to moderate kinematic correlations for some joints, the joint moments required to produce the respective motions exhibited similar trends. The two greatest differences observed between correlation values of kinematics relative to kinetics were at the knee adduction joint (kinematic correlation = 0.45, kinetic correlation = 0.61, delta = 0.16) and the ankle subtalar joint (kinematic correlation = 0.06, kinetic correlation = 0.74, delta = 0.68).

In conclusion, markerless motion capture systems have the potential to vastly expand the impact of 3D motion analysis used across the many patient and athlete populations. This study provides additional insight into the utility of using a markerless system to measure lower extremity kinematics and kinetics. Further verification of markerless-derived biomechanical data will help demonstrate validity and practicality in translation settings. Development and validation of markerless systems will lead to more ubiquitous, routine quantification of biomechanics in non-laboratory settings (e.g., clinical environments, rehabilitation settings). In addition, investigation into the benefit of using synthetic training data in other environments to further improve markerless motion capture system performance is warranted.

## Data Availability

The datasets presented in this article are not readily available because these data are part of a larger clinical trial that is not completed or published. Once published, the data could be made available by request. Requests to access the datasets should be directed to ty.templin@swri.org.

## References

[B1] Alcantara R. S. Edwards W. B. Millet G. Y. Grabowski A. M. (2022). Predicting continuous ground reaction forces from accelerometers during uphill and downhill running: a recurrent neural network solution. PeerJ 10, e12752. 10.7717/peerj.12752 35036107 PMC8740512

[B2] Andriluka M. Pishchulin L. Gehler P. Schiele B. (2014). “2D human pose estimation: new benchmark and state of the art analysis,” in 2014 IEEE conference on computer vision and pattern recognition.

[B3] Bates N. A. Ford K. R. Myer G. D. Hewett T. E. (2013). Kinetic and kinematic differences between first and second landings of a drop vertical jump task: implications for injury risk assessments. Clin. Biomech. 28 (4), 459–466. 10.1016/j.clinbiomech.2013.02.013 PMC3809751 23562293

[B4] Benoit D. L. Damsgaard M. Andersen M. S. (2015). Surface marker cluster translation, rotation, scaling and deformation: their contribution to soft tissue artefact and impact on knee joint kinematics. J. Biomechanics 48 (10), 2124–2129. 10.1016/j.jbiomech.2015.02.050 25935684

[B5] Benoit D. L. Ramsey D. K. Lamontagne M. Xu L. Wretenberg P. Renström P. (2006). Effect of skin movement artifact on knee kinematics during gait and cutting motions measured *in vivo* . Gait and Posture 24 (2), 152–164. 10.1016/j.gaitpost.2005.04.012 16260140

[B6] Cao Z. Hidalgo G. Simon T. Wei S.-E. Sheikh Y. (2018). OpenPose: realtime multi-person 2D pose estimation using Part Affinity fields. arXiv:1812.08008. 10.1109/TPAMI.2019.2929257 31331883

[B7] Cappozzo A. Catani F. Leardini A. Benedetti M. Della Croce U. (1996). Position and orientation in space of bones during movement: experimental artefacts. Clin. Biomech. 11 (2), 90–100. 10.1016/0268-0033(95)00046-1 11415604

[B8] Delp S. L. Anderson F. C. Arnold A. S. Loan P. Habib A. John C. T. (2007). OpenSim: open-source software to create and analyze dynamic simulations of movement. IEEE Trans. Biomed. Eng. 54 (11), 1940–1950. 10.1109/tbme.2007.901024 18018689

[B9] DiCesare C. A. Montalvo A. Foss K. D. Thomas S. M. Hewett T. E. Jayanthi N. A. (2019). Sport specialization and coordination differences in multisport adolescent female basketball, soccer, and volleyball athletes. J. Athl. Train. 54 (10), 1105–1114. 10.4085/1062-6050-407-18 31633418 PMC6805056

[B10] Fischler M. A. Bolles R. C. (1981). Random sample consensus. Commun. ACM 24 (6), 381–395. 10.1145/358669.358692

[B11] Ford K. R. Myer G. D. Brent J. L. Hewett T. E. (2009). Hip and knee extensor moments predict vertical jump height in adolescent girls. J. Strength Cond. Res. 23 (4), 1327–1331. 10.1519/jsc.0b013e31819bbea4 19528842 PMC4010199

[B12] Ford K. R. Myer G. D. Hewett T. E. (2003). Valgus knee motion during landing in high school female and male basketball players. Med. and Sci. Sports and Exerc. 35 (10), 1745–1750. 10.1249/01.mss.0000089346.85744.d9 14523314

[B13] Ford K. R. Myer G. D. Hewett T. E. (2007). Reliability of landing 3D motion analysis: implications for longitudinal analyses. Med. and Sci. Sports and Exerc. 39 (11), 2021–2028. 10.1249/mss.0b013e318149332d 17986911

[B14] Ford K. R. Myer G. D. Hewett T. E. (2010). Longitudinal effects of maturation on lower extremity joint stiffness in adolescent athletes. Am. J. Sports Med. 38 (9), 1829–1837. 10.1177/0363546510367425 20522830 PMC3968426

[B15] Ford K. R. Myer G. D. Smith R. L. Byrnes R. N. Dopirak S. E. Hewett T. E. (2005). Use of an overhead goal alters vertical jump performance and biomechanics. J. Strength Cond. Res. 19 (2), 394. 10.1519/15834.1 15903381

[B16] Ford K. R. Shapiro R. Myer G. D. Van Den Bogert A. J. Hewett T. E. (2010). Longitudinal sex differences during landing in knee abduction in young athletes. Med. and Sci. Sports and Exerc. 42 (10), 1923–1931. 10.1249/mss.0b013e3181dc99b1 20305577 PMC2924455

[B17] Gilbert A. Trumble M. Malleson C. Hilton A. Collomosse J. (2019). Fusing visual and inertial sensors with semantics for 3D human pose estimation. Int. J. Comput. Vis. 127 (4), 381–397. 10.1007/s11263-018-1118-y

[B18] Gorton G. E. Hebert D. A. Gannotti M. E. (2009). Assessment of the kinematic variability among 12 motion analysis laboratories. Gait and Posture 29 (3), 398–402. 10.1016/j.gaitpost.2008.10.060 19056271

[B19] Hewett T. E. Myer G. D. Ford K. R. (2004). Decrease in neuromuscular control about the knee with maturation in female athletes. J. Bone Jt. Surgery-American Volume 86 (8), 1601–1608. 10.2106/00004623-200408000-00001 15292405

[B20] Hewett T. E. Myer G. D. Ford K. R. (2005a). Reducing knee and anterior cruciate ligament injuries among female athletes: a systematic review of neuromuscular training interventions. J. Knee Surg. 18 (1), 82–88. 10.1055/s-0030-1248163 15742602

[B21] Hewett T. E. Myer G. D. Ford K. R. Heidt R. S. Colosimo A. J. McLean S. G. (2005b). Biomechanical measures of neuromuscular control and valgus loading of the knee predict anterior cruciate ligament injury risk in female athletes: a prospective study. Am. J. Sports Med. 33 (4), 492–501. 10.1177/0363546504269591 15722287

[B22] Hewett T. E. Roewer B. Ford K. Myer G. (2015). Multicenter trial of motion analysis for injury risk prediction: lessons learned from prospective longitudinal large cohort combined biomechanical - epidemiological studies. Rev. Bras. Fisioter. 19 (5), 398–409. 10.1590/bjpt-rbf.2014.0121 PMC4647151 26537810

[B23] Ionescu C. Papava D. Olaru V. Sminchisescu C. (2014). Human3.6M: large scale datasets and predictive methods for 3D human sensing in natural environments. IEEE Trans. Pattern Analysis Mach. Intell. 36 (7), 1325–1339. 10.1109/tpami.2013.248 26353306

[B24] Ito N. Sigurðsson H. B. Seymore K. D. Arhos E. K. Buchanan T. S. Snyder-Mackler L. (2022). Markerless motion capture: what clinician-scientists need to know right now. JSAMS Plus 1, 100001. 10.1016/j.jsampl.2022.100001 36438718 PMC9699317

[B25] Johnson S. Everingham M. (2010). “Clustered pose and nonlinear appearance models for human pose estimation,” in British machine vision conference.

[B26] Johnson W. R. Mian A. Donnelly C. J. Lloyd D. Alderson J. (2018). Predicting athlete ground reaction forces and moments from motion capture. Med. and Biol. Eng. and Comput. 56 (10), 1781–1792. 10.1007/s11517-018-1802-7 29550963

[B27] Kanko R. M. Laende E. K. Davis E. M. Selbie W. S. Deluzio K. J. (2021). Concurrent assessment of gait kinematics using marker-based and markerless motion capture. J. Biomechanics 127, 110665. 10.1016/j.jbiomech.2021.110665 34380101

[B28] Kanko R. M. Outerleys J. B. Laende E. K. Selbie W. S. Deluzio K. J. (2024). Comparison of concurrent and asynchronous running kinematics and kinetics from marker-based and markerless motion capture under varying clothing conditions. J. Appl. Biomechanics 40, 129–137. 10.1123/jab.2023-0069 38237574

[B29] Kessler S. E. Rainbow M. J. Lichtwark G. A. Cresswell A. G. D'Andrea S. E. Konow N. (2019). A direct comparison of biplanar videoradiography and optical motion capture for foot and ankle kinematics. Front. Bioeng. Biotechnol. 7, 199. 10.3389/fbioe.2019.00199 31508415 PMC6716496

[B30] Leardini A. Chiari L. Croce U. D. Cappozzo A. (2005). Human movement analysis using stereophotogrammetry. Gait and Posture 21 (2), 212–225. 10.1016/j.gaitpost.2004.05.002 15639400

[B31] Lin T.-Y. Maire M. Belongie S. Hays J. Perona P. Ramanan D. (2014). Microsoft COCO: Common Objects in Context. Springer International Publishing, 740–755. 10.1007/978-3-319-10602-1_48

[B32] Louis N. Templin T. N. Eliason T. D. Nicolella D. P. Corso J. J. (2022). Learning to estimate external forces of human motion in video. arXiv:2207.05845. 10.1145/3503161.3548377 PMC13073551 41982740

[B33] Lucchetti L. Cappozzo A. Cappello A. Croce U. D. (1998). Skin movement artefact assessment and compensation in the estimation of knee-joint kinematics. J. Biomechanics 31 (11), 977–984. 10.1016/s0021-9290(98)00083-9 9880054

[B34] Miller J. D. Cabarkapa D. Miller A. J. Frazer L. L. Templin T. N. Eliason T. D. (2023). Novel 3D force sensors for a cost-effective 3D force plate for biomechanical analysis. Sensors 23 (9), 4437. 10.3390/s23094437 37177650 PMC10181757

[B35] Miranda D. L. Rainbow M. J. Crisco J. J. Fleming B. C. (2013). Kinematic differences between optical motion capture and biplanar videoradiography during a jump–cut maneuver. J. Biomechanics 46 (3), 567–573. 10.1016/j.jbiomech.2012.09.023 PMC3551998 23084785

[B36] Miranda D. L. Schwartz J. B. Loomis A. C. Brainerd E. L. Fleming B. C. Crisco J. J. (2011). Static and dynamic error of a biplanar videoradiography system using marker-based and markerless tracking techniques. J. Biomechanical Eng. 133, 121002. 10.1115/1.4005471 PMC3267989 22206419

[B37] Myer G. D. Ford K. R. Khoury J. Hewett T. E. (2011). Three-dimensional motion analysis validation of a clinic-based nomogram designed to identify high ACL injury risk in female athletes. Physician Sports Med. 39, 19–28. 10.3810/psm.2011.02.1838 PMC9896426 21378483

[B38] Myer G. D. Ford K. R. Palumbo J. P. Hewett T. E. (2005). Neuromuscular training improves performance and lower-extremity biomechanics in female athletes. J. Strength Cond. Res. 19 (1), 51. 10.1519/13643.1 15705045

[B39] Myer G. D. Stroube B. W. Dicesare C. A. Brent J. L. Ford K. R. Heidt R. S. (2013). Augmented feedback supports skill transfer and reduces high-risk injury landing mechanics. Am. J. Sports Med. 41 (3), 669–677. 10.1177/0363546512472977 23371471 PMC4166501

[B40] Myer G. D. Wordeman S. C. Sugimoto D. Bates N. A. Roewer B. D. Medina McKeon J. M. (2014). Consistency of clinical biomechanical measures between three different institutions: implications for multi-center biomechanical and epidemiological research. Int. J. sports Phys. Ther. 9 (3), 289–301. 24944847 PMC4060306

[B41] Nakano M. Kubota K. Hashizume S. Kobayashi E. Chikenji T. S. Saito Y. (2020). An enriched environment prevents cognitive impairment in an Alzheimer’s disease model by enhancing the secretion of exosomal microRNA-146a from the choroid plexus. Brain, Behav. and Immun. - Health 9, 100149. 10.1016/j.bbih.2020.100149 PMC8474441 34589894

[B42] Needham L. Evans M. Wade L. Cosker D. P. McGuigan M. P. Bilzon J. L. (2022). The development and evaluation of a fully automated markerless motion capture workflow. J. Biomechanics 144, 111338. 10.1016/j.jbiomech.2022.111338 36252308

[B43] Patel P. Huang C.-H. P. Tesch J. Hoffmann D. T. Tripathi S. Black M. J. (2021). AGORA: avatars in geography optimized for regression analysis. arXiv:2104.14643.

[B44] Paterno M. V. Schmitt L. C. Ford K. R. Rauh M. J. Myer G. D. Huang B. (2010). Biomechanical measures during landing and postural stability predict second anterior cruciate ligament injury after anterior cruciate ligament reconstruction and return to sport. Am. J. Sports Med. 38 (10), 1968–1978. 10.1177/0363546510376053 20702858 PMC4920967

[B45] Pavlakos G. Choutas V. Ghorbani N. Bolkart T. Osman A. A. A. Tzionas D. (2019). Expressive body capture: 3D hands, face, and body from a single image. arXiv:1904.05866.

[B46] Pedley J. S. Lloyd R. S. Read P. J. Moore I. S. De Ste Croix M. Myer G. D. (2020). Utility of kinetic and kinematic jumping and landing variables as predictors of injury risk: a systematic review. J. Sci. Sport Exerc. 2 (4), 287–304. 10.1007/s42978-020-00090-1

[B47] Peters A. Galna B. Sangeux M. Morris M. Baker R. (2010). Quantification of soft tissue artifact in lower limb human motion analysis: a systematic review. Gait and Posture 31 (1), 1–8. 10.1016/j.gaitpost.2009.09.004 19853455

[B48] Rajagopal A. Dembia C. L. Demers M. S. Delp D. D. Hicks J. L. Delp S. L. (2016). Full-body musculoskeletal model for muscle-driven simulation of human gait. IEEE Trans. Biomed. Eng. 63 (10), 2068–2079. 10.1109/tbme.2016.2586891 27392337 PMC5507211

[B49] Reinschmidt C. Van Den Bogert A. J. Nigg B. M. Lundberg A. Murphy N. (1997). Effect of skin movement on the analysis of skeletal knee joint motion during running. J. Biomechanics 30 (7), 729–732. 10.1016/s0021-9290(97)00001-8 9239553

[B50] Roewer B. Ford K. Myer G. Hewett T. (2014). The ‘impact’ of force filtering cut-off frequency on the peak knee abduction moment during landing: artefact or ‘artifiction’? Br. J. Sports Med. 48 (6), 464–468. 10.1136/bjsports-2012-091398 22893510 PMC4163685

[B51] Schober P. Boer C. Schwarte L. A. (2018). Correlation coefficients: appropriate use and interpretation. Anesth. Analg. 126, 1763–1768. 10.1213/ane.0000000000002864 29481436

[B52] Sigal L. Balan A. O. Black M. J. (2010). HumanEva: synchronized video and motion capture dataset and baseline algorithm for evaluation of articulated human motion. Int. J. Comput. Vis. 87 (1-2), 4–27. 10.1007/s11263-009-0273-6

[B53] Sigal L. Sclaroff S. Athitsos V. (2004). Skin color-based video segmentation under time-varying illumination. IEEE Trans. Pattern Analysis Mach. Intell. 26 (7), 862–877. 10.1109/tpami.2004.35 18579945

[B54] Song K. Hullfish T. J. Scattone Silva R. Silbernagel K. G. Baxter J. R. (2023). Markerless motion capture estimates of lower extremity kinematics and kinetics are comparable to marker-based across 8 movements. J. Biomechanics 157, 111751. 10.1016/j.jbiomech.2023.111751 PMC10494994 37552921

[B55] Strutzenberger G. Kanko R. Selbie S. Schwameder H. Deluzio K. (2021). Assessment of kinematic CMJ data using a deep learning algorithm-based markerless motion capture system. ISBS Proc. Arch. 39. Iss. 1, Article 61. 10.1016/j.jbiomech.2021.110414 33915475

[B56] Tang H. Pan J. Munkasy B. Duffy K. Li L. (2022). Comparison of lower extremity joint moment and power estimated by markerless and marker-based systems during treadmill running. Bioengineering 9 (10), 574. 10.3390/bioengineering9100574 36290542 PMC9598493

[B57] Templin T. Eliason T. Medjaouri O. Chambers D. Saylor K. Nicolella D. (2023). The effect of synthetic training data on the performance of a deep learning based markerless biomechanics system. bioxRiv. 10.1101/2023.10.19.562758

[B58] Uhlrich S. D. Falisse A. Kidziński Ł. Muccini J. Ko M. Chaudhari A. S. (2023). OpenCap: human movement dynamics from smartphone videos. PLOS Comput. Biol. 19 (10), e1011462. 10.1371/journal.pcbi.1011462 37856442 PMC10586693

[B59] Varol G. Romero J. Martin X. Mahmood N. Black M. J. Laptev I. (2017). Learning from synthetic humans. arXiv:1701.01370.

[B60] Weitz A. Colucci L. Primas S. Bent B. (2021). InfiniteForm: a synthetic, minimal bias dataset for fitness applications. arXiv:2110.01330.

[B61] Xu D. Zhou H. Quan W. Gusztav F. Wang M. Baker J. S. (2023). Accurately and effectively predict the ACL force: utilizing biomechanical landing pattern before and after-fatigue. Comput. Methods Programs Biomed. 241, 107761. 10.1016/j.cmpb.2023.107761 37579552

[B62] Xu D. Zhou H. Quan W. Jiang X. Liang M. Li S. (2024). A new method proposed for realizing human gait pattern recognition: inspirations for the application of sports and clinical gait analysis. Gait and Posture 107, 293–305. 10.1016/j.gaitpost.2023.10.019 37926657

